# Integrated Care: A Pill for All Ills?

**DOI:** 10.15171/ijhpm.2016.111

**Published:** 2016-08-13

**Authors:** Maria Goddard, Anne R. Mason

**Affiliations:** Centre for Health Economics, University of York, York, UK.

**Keywords:** Integrated Care, Health Sector, Social Care Sector

## Abstract

There is an increasing policy emphasis on the integration of care, both within the healthcare sector and also between the health and social care sectors, with the simple aim of ensuring that individuals get the right care, in the right place, at the right time. However, implementing this simple aim is rather more complex. In this editorial, we seek to make sense of this complexity and ask: what does integrated care mean in practice? What are the mechanisms by which it is expected to achieve its aim? And what is the nature of the evidence base around the outcomes delivered?

## Introduction

Described as a “global buzzword in healthcare,” integrated care is widely viewed as offering a potential solution to some of the major challenges facing health systems across the world.^1^ In high-income and low- and middle-income countries,^2,3^ as the pressures on budgets grow, integrated care is increasingly invoked as a way forward for addressing both financial and quality issues by tackling fragmentation, duplication and poor co-ordination of care. As a consequence, major policy developments in the organisation and financing of care have been driven by the integration agenda. However, it is often unclear what is actually meant by the “elastic concept”^4^ of integrated care; how the concept translates to integration in practical terms; how it is expected to achieve the stated objectives; and the nature of the evidence base to support it.

## What Is Integrated Care?

It is clear from the literature that “integrated care” is used as an umbrella term for a wide variety of concepts and organisational structures. In order to assess whether variants of integrated care have the potential to deliver on ambitious policy aims, clarity is required about what goes on under the banner of integration. Conceptually, consensus suggests that the starting point in defining integration should be to focus on patients and citizens, rather than on structures and organisations. Thus, it has sometimes been defined as a means of delivering enhanced access to care and improved quality of care, especially for those with complex problems whose needs cut across multiple services, providers and settings.^4^ Similarly, it has been described as “person-centred, coordinated, and tailored to the needs and preferences of the individual, their carer and family…and puts the needs and experience of people at the centre of how services are organised and delivered.”^5^

In terms of the practical arrangements that underpin these broad principles, there are also a number of different ways in which integration can be represented. It can be thought of in terms of three “levels” defined by the group to whom care is delivered.^6^ The macro level at which providers deliver integrated care across the full spectrum of services to the entire population (eg, Kaiser Permanente, the Veterans Health Administration and other types of accountable care organisations); second, the meso level, where care is delivered to particular sub-populations (eg, older people, mental health); and third, the micro level where care is delivered to individual service users and their carers (eg, through care co-ordination and planning). Alternatively, integrated care may be defined with reference to the dimensions along which it occurs eg, integration of administrative/functional aspects of services, or of clinical processes; financial responsibility, cultural and professional values; or to the breadth of integration: horizontal integration between organisations operating at the same level in the supply chain either within one sector (eg, two hospitals), or across sectors (eg, health and social care staff working in one setting); and vertical integration between providers at different points in the care pathway (eg, hospital and community providers; primary and secondary care). Vertical integration may also refer to integration between providers and commissioners/payers of care which may be confined within the healthcare system or may cut across sectors, involving both health and social care providers. A distinction also arises between the degree of integration, for example, so-called virtual integration where organisations work together via networks and alliances or “real” integration, where organisations physically merge premises and staff groups. It is not possible within the scope of this paper to define the concepts and the entire set of potential integrated care arrangements - the “imprecise hodgepodge” of meanings has been noted elsewhere.^1^ But this brief overview suggests that while there may well be a broadly shared understanding of the very general principles underpinning integrated care, the way in which it is implemented in practice, and the detail of the arrangements of made on the ground, is likely to vary enormously. This has important implications for the evaluation of integrated care: in order to understand how it might achieve benefits and indeed, whether the benefits are realised, we need to keep sight of the general principles and the degree to which they are adhered to, whilst ensuring there is also clarity about the precise nature of how integrated care is operationalised and implemented.

## How Can Integrated Care Achieve its Objectives?

The potential impacts of integrated care have been summarised elsewhere^7-9^ and include better access, improved satisfaction and experience for patients, carers and health professionals, more appropriate care, enhanced preventive care, reduced avoidable hospital admissions and emergency admissions, prolonged independent living and delayed admission to institutional care, improved health status and quality of life, enhanced cost-effectiveness.

A wide range of “enablers” that may help to achieve these objectives have been identified in the literature (eg, Cameron et al,^10^ summarises). These include:

- Professional and cultural enablers such as: a common purpose and vision, shared professional values, shared culture, strong leadership, joint working, trust and a willingness to look beyond the interests of single organisations.
- Organisational enablers such as: involvement of staff at all levels, clarity about roles and responsibilities, ability to share data across organisations, lack of legal obstacles, incentives for collaboration, mechanisms for dealing with different locations of providers, clarity on financial arrangements, sufficient funding to support integration, trained and engaged workforce.
- Policy enablers such as: payment mechanisms that support cross-organisational care, consistent regulatory policies, and the political will to support appropriate structural changes.

The relative importance of these factors in contributing to the success of integrated care will vary depending upon the model of integration – for instance, shared professional values may be easier to achieve when integrating services within the health sector as opposed to across the health and social care sectors. Other factors may in principle be particularly powerful, for instance, the lack of financial integration has been identified as a major barrier to the success of many integrated care schemes.^11^ Hence, mechanisms that achieve financial integration across organisational or sector boundaries should align provider objectives, support coordinated care, reduce incentives to cost shift and encourage efficiency.^12^ Indeed, the key role of financial integration has underpinned some of the most recent developments in the English health and social care sectors, including the creation of the Better Care Fund which provides for pooled budgets across health and social care, used to fund integration plans from 2014.^13^ As part of the strategy to develop new models of care that will improve quality and also release pressure on resources, national initiatives have been introduced. These include the Integrated Care and Support Pioneer programme, and, more recently, the so-called Vanguard sites. All the new models and initiatives seek to develop and test new models of integrated care for a range of patient groups and care settings.^14,15^ Some of these new approaches have the potential to go beyond the integration of organisations as they attempt to achieve “whole system” integration across hospital, community, primary and secondary care, facilitated in some cases by a single capitated budget. These schemes focus on populations, places and systems and are similar in nature to the “accountable care organisations” developed in the United States.^16^

## What Does the Evidence Tell us?

Although there is an extensive literature, robust evaluations – in particular, randomised controlled trials – of integrated care programmes are rare. This is partly because they are challenging methodologically and, as a result, uncontrolled before and after comparisons and patient/staff surveys, are a common approach. It is, therefore, easy to be convinced of the value of the approach without questioning the basis of the evidence. Interpretation and generalisation of the evidence from specific schemes is also difficult due to the wide variation in the meaning of the terminology, both conceptually and in practice, as outlined earlier.

The overall message emerging from most careful evaluations is that the evidence on benefits is rather mixed. A review of schemes of integrated care along the “micro to macro” scale, provided some examples of good practice, but overall was unable to point to a specific approach that delivered the full range of expected benefits.^6^ Others have noted that particular types of integration – vertical integration for instance – can produce benefits in terms of building partnerships between organisations and services, but the evidence-based “remains weak” in relation to patient experience, clinical outcomes and costs and that there are “significant gaps” in the evidence related to key measures of the impact of integration.^17^ A review of the international evidence relating to integrated care schemes across health and social care that incorporated financial integration identified 38 schemes which, in theory, should provide powerful incentives for change, but concluded that “the case for integrated funding has not yet been demonstrated.”^11^ No scheme achieved a sustained reduction in hospital use, although there was some evidence that access to community services could be improved. Only a small fraction of the schemes delivered significant improvements in health outcomes and in only 3 cases was there evidence of a significant reduction in utilisation or costs. Financial integration is at the heart of some of the new models of care currently underway in England, organised along the lines of the “accountable care organisation” model, although again the early evidence on the impact of these in the United States, is quite limited and is mixed.^18^

The findings relating to utilisation and costs highlight one of the major issues in considering the strategic shift towards integrated care in many countries: it is often viewed as a means of generating cost savings - by focusing on preventive care, shifting care out of more expensive secondary care sectors into the community, and by reducing avoidable admissions. However, this may be more of a hope than a fact. An evaluation of the Pioneer schemes in England^19^ suggests that one major issue emerging is that shifting care out of acute hospitals will only produce a saving if beds are closed, which is a radical path for most providers to follow and deeply unpopular with the public, even if it is warranted on economic or quality grounds. Where provider income is tied to activity rates, changing behaviour is a challenging task. The evidence base on cost savings from the Pioneers was deemed to be “deficient” and many were sceptical that providing care in the community is actually less costly than hospital care. Even where such changes do occur for individuals, the nature of integrated care means that overall system costs may increase. A model that focuses greater attention on patient needs increases the likelihood that coordinated care ‘reveals rather than resolves’ unmet need.^20^ Whilst identifying and treating unmet needs may be a beneficial outcome for society, its feasibility depends upon policy-makers’ willingness and ability to pay.

## Conclusion

This brief overview suggests that the “language” of integrated care can be quite general, but it is important to be clear from a conceptual and practical perspective what is meant by the terms employed. The potential benefits for patients and for the health and care system are significant in principle, but the breadth, intensity and nature of integration will determine the degree to which such benefits can be reaped in practice. Key elements of the process, such as the degree of financial integration and the alignment of vision and cultures across professional boundaries, will influence the capacity of integrated care to deliver its potential. Last, the evidence base is not definitive and there are many gaps in knowledge about the degree to which most of the expected benefits from integrated care will materialise: “integrated organizational structures and processes may fail to produce integrated patient care.”^21^

Doubts about the ability of integrated care to deliver cost savings across the health and care system are especially acute. Many such systems are currently operating in a challenging financial environment, which is in itself one of the motivating factors for the pursuit of integrated care solutions. However, an “integration paradox”^19^ may emerge whereby the incentives for organisations to collaborate are mitigated by the perceived need to protect existing activities and resources that currently rest within organisational boundaries, precisely because of the harsh financial climate in which they are currently operating.

Whilst the principles underpinning integrated care are simple and uncontroversial – a mechanism for providing the right care in the right place at the right time – the implementation is much more complex.^22^ There is a growing body of knowledge about the enablers and barriers to integrated care, but it is clear that identifying the right sort of “ingredients” does not in itself guarantee that they will deliver the right outcomes in practice. Understanding the specific context for integrated care and the actual “craft and graft” employed by the workforce tasked with delivering integration in reality, is equally as important as understanding the science.^23^ The evidence suggests that what happens in practice is rarely the optimal arrangement planned at the outset and thus, testing whether a fully integrated care system is really a “pill for all ills” is stymied by the significant implementation problem.

## Ethical issues

Not applicable.

## Competing interests

Authors declare that they have no competing interests.

## Authors’ contributions

Both authors contributed substantially to the conception of the paper. MG wrote the first draft and AM critically revised it.

## References

[1] Kodner D. All together now: a conceptual exploration of integrated care. Healthcare Q 2009; 2009;13 Spec No:6-15. 10.12927/hcq.2009.21091 20057243

[2] Goodwin N, Smith J, Davies A, et al. A Report to the Department of Health and the NHS Future Forum – Integrated care for patients and populations: Improving outcomes by working together. London: The King’s Fund and Nuffield Trust; 2012.

[3] Lê G, Morgan R, Bestall J, Featherstone Featherstone, Veale T, Ensor T (2016). Can service integration work for universal health coverage? Evidence from around the globe. Health Policy.

[4] Kodner DL, Spreeuwenberg C (2002). Integrated care: meaning, logic, application, and implications – a discussion paper. Int J Integr Care.

[5] National Collaboration for Integrated Care and Support. Integrated care and Support: our shared commitment. London: Department of Health; 2013.

[6] Ham C, Curry N. Integrated care – What is it? Does it work? What does it mean for the NHS? London: The King’s Fund; 2011.

[7] Nolte E, Pitchforth E. What is the evidence on the economic impacts of integrated care? Policy Summary 11. Copenhagen: World Health Organisation; 2014.

[8] Ham C, Curry N. Clinical and service integration: the route to improved outcomes. London: The King’s Fund; 2010.

[9] Mason A, Goddard M, Weatherly H. Financial mechanisms for integrating funds for health and social care: an evidence review. CHE Research Paper No. 97. York: University of York, Centre for Health Economics; 2014.

[10] Cameron A, Lart, R, Bostock L, Coomber C. Factors that promote and hinder joint and integrated working between health and social care services. Research Briefing 41. London: Social Institute for Excellence; 2012. 10.1111/hsc.12057 23750908

[11] Mason A, Goddard M, Weatherly H, Chalkley M (2015). Integrating funds for health and social care: an evidence review. J Health Serv Res Policy.

[12] Audit Commission. Means to an end: Joint financing across health and social care. London: Audit Commission; 2009.

[13] Department of Health, Department for Communities and Local Government. Better Care Fund, Policy Framework 2016/17. London: Department of Health; 2016.

[14] NHS England. People helping people. Year two of the pioneer programme. London: NHS England Publication Gateway Ref 04386; 2016.

[15] NHS England. New care models: Vanguards – developing a blueprint for the future of NHS and care services. London: NHS England; 2016.

[16] Ham C, Alderwick H. Place-based systems of care: A way forward for the NHS in England. London: The King’s Fund 2015.

[17] Ramsay A, Fulop N, Edwards N (2009). The evidence base for vertical integration in health care. J Integr Care.

[18] Shortell S (2015). The NHS five year forward view: lessons from the United States in developing new care models. BMJ.

[19] Erens B, Wistow G, Mounier-Jack S, et al. Early evaluation of the Integrated Care and Support Pioneers Programme. Policy Innovation Research Unit (PIRU); 2016-2017.

[20] Segal L, Dunt D, Day SE (2004). Introducing coordinated care (2): evaluation of design features and implementation processes implications for a preferred health system reform model. Health Policy.

[21] Singer S, Burgers J, Friedberg M, Rosenthal MB, Leape L, Schneider E (2011). Defining and measuring integrated patient care: promoting the next frontier in health care delivery. Med Care Res Rev.

[22] Bardsley M, Steventon A, Smith J, Dixon J. Evaluating integrated and community-based care – How do we know what works? Research summary. London: Nuffield Trust; 2013.

[23] Dickinson H (2014). Making a reality of integration: less science, more craft and graft. J Integr Care.

